# Dietary protein levels impact growth, meat quality, and muscle protein pathways in growing-finishing pigs under low-temperature conditions

**DOI:** 10.5713/ab.260002

**Published:** 2026-06-01

**Authors:** Ting Xie, Zhibo Wu, Feng Li, Zhongyu Wang, Tianshu Liu, Nengwen Liu, Mengmeng Sun, Mingwei Song, Na Dong

**Affiliations:** 1 College of Animal Science and Technology, Northeast Agricultural University, Harbin, China

**Keywords:** Crude Protein, Growth Performance, Low Temperature, Meat Quality, Muscle Protein Synthesis

## Abstract

**Objective:**

Appropriately increasing dietary protein levels during winter production can improve meat quality and enhance nutritional value. Here, we examined the effects of dietary protein levels under low-temperature conditions (14°C) on growth performance, meat quality, and protein synthesis pathways in growing-finishing pigs.

**Methods:**

Forty-eight pigs were randomly assigned to four treatments (T1, T2, T3 and T4) with protein levels of 15.93%, 14.92%, 13.56%, and 11.36% for each treatment. Protein level of T2, T3, and T4 was 104%, 108%, and 112% of T1, respectively. Growth performances were studied at four weitht stages (25–50, 50–75, 75–100, 100–125 kg).

**Results:**

Throughout the entire growth period, T2 shortened the production cycle and exhibited the lowest feed/gain ratio. It also significantly improved the meat quality characteristics of the longissimus thoracis muscle (p<0.05), as evidenced by reduced drip loss and cooking loss, alongside increased protein and amino acid content. Additionally, T2 and T4 upregulated *IGF-1* and *SLC38A2* gene expression, while elevating p-mTOR, p-S6K1, and p-4E-BP1 protein levels to enhance protein synthesis. Although T4 enhanced meat nutritional value, it resulted in reduced early growth performance. However, upregulation of *MuRF1* gene expression in T3 indicated accelerated protein degradation processes.

**Conclusion:**

In this study, increasing dietary protein levels to 104% under low temperatures shortened the swine production cycle and improved meat quality and nutritional value. This regulatory effect may be associated with the activation of the mTOR signaling pathway.

## INTRODUCTION

As energy prices continue to rise worldwide, an increasing number of pig farmers are adopting measures to reduce heating in winter and implementing low-temperature breeding models to save energy consumption. Pigs are particularly sensitive to temperature fluctuations, with the critical temperature range typically considered to be 26°C–28°C for weaned piglets and 18°C–20°C for finishing pigs [1]. Consequently, lowering the temperature in pig barns not only increases gastrointestinal motility and reduces feed efficiency, affecting growth performance, but also alters muscle metabolism, thereby increasing the risk of inferior meat quality [2]. Therefore, when adopting lower rearing temperatures for energy conservation, reliance on the pigs’ own regulatory mechanisms alone is insufficient. It is essential to optimize nutritional supply to enhance growth performance in cold environments and mitigate negative impacts on meat quality. As an essential nutrient in animal diets, protein significantly contributes to regulating physiological activities, and enhancing muscle protein synthesis and supporting flavor amino acid (FAA) deposition [3,4]. Protein metabolism encompasses both the synthesis pathway mediated by the mammalian target of rapamycin (mTOR) and the degradation pathway mediated by the ubiquitin-proteasome system. mTOR regulates protein synthesis by activating downstream factors, primarily promoting protein synthesis and deposition in animal muscle through the phosphorylation of ribosomal protein S6 kinase 1 (S6K1) and eukaryotic translation initiation factor 4E binding protein 1 (4E-BP1) [5]. Conversely, MuRF1 and MAFbx as E3 ubiquitin ligases promote degradation together with FOXO [6]. We hypothesize that appropriate protein intake may help pigs alleviate protein metabolic disorders and cold-induced meat quality deterioration via the mTOR pathway, while also improving their growth performance. Currently, few studies have investigated dietary protein supplementation under low-temperature conditions. In this study, feeding trials with graded dietary protein levels under cold conditions in winter to explore the effects of dietary protein concentration on growing-finishing pigs’ growth performance, meat quality, and nutritional quality, as well as their underlying regulatory mechanisms.

## MATERIALS AND METHODS

### Animal care

This research was carried out at the breeding site of Northeast Agricultural University in Acheng.

### Animals and diets

Forty-eight Duroc×Landrace×Yorkshire pigs (24.65±0.34 kg) were randomly assigned to 4 dietary treatments, with 12 pigs per treatment (T1, T2, T3, T4) and individual replication as the experimental unit and genetic background consistency was controlled to minimize intergroup differences. All pigs were housed in the same finishing barn; the pens for the four treatment groups were located in the center and adjacent to one another, with consistent environmental conditions and management practices maintained across all groups. The trial had four stages by pig weight (25–50, 50–75, 75–100, 100–125 kg). T1’s dietary protein levels at each stage were 15.93%, 14.92%, 13.56%, 11.36% based on the Nutritional Requirements of Swine (NRS, GB/T 39235-2020) [7], while T2/T3/T4 were 104%/108%/112% of T1 respectively at each stage. Amino acid (AA) levels in the diets were increased proportionally to the protein levels. To meet the increased maintenance energy requirements resulting from the low-temperature environment, the net energy level of each diet stage was increased by 6% above the recommended level of NRS [8]. The diets of each treatment group were shown in Supplement 1. The pig house temperature was maintained at 14°C, and the experimental protocol ensured pigs could consume feed and water freely.

### Growth performance

Throughout the experiment, an intelligent pig identification system (WJ-4; Beijing Wangjing Muyuan Technology) was used to automatically identify each pig via electronic ear tags and record real-time data on feed intake and body weight for each pig, ensuring that these measurements were independent for each individual pig. Based on this data, average daily gain (ADG), average daily feed intake (ADFI), and feed/gain (F/G) were calculated.

### Sample collection

At the end of the feeding trial, pigs were fasted for 12 h. Six pigs per group were randomly selected for blood collection via the anterior vena cava. Blood samples were centrifuged at 3,000×g for 15 min and stored at −20°C. Subsequently, painless electrocution slaughter was performed. Postmortem, the longissimus thoracis (LT) muscle between the 10th and 11th thoracic vertebrae on the left side was collected and frozen at −80°C for molecular biology testing. LT samples collected from the corresponding right-side locations were stored at 4°C for subsequent meat quality analysis.

### Meat quality measurements

A pH meter with automatic temperature compensation (PB-10; Sartorius) was used to determine the LT’s pH at 45 min postmortem. The pH value of the muscle samples was measured after being stored at 4°C for 24 h. The pH meter calibration was performed (4°C) using commercial standard buffers with pH of 4.0 and 7.0. At 45 min postmortem, a colorimeter (CR-400; Konica Minolta) was used to measure the L* (brightness), a* (redness), and b* (yellowness) of LT (8 mm aperture, D65 illuminant, 8° observer angle). The color parameters of the meat samples were re-measured after being stored at 4°C for 24 h. About 40 g of LT was excised and suspended in a plastic bottle with a fishing line, allowing it to drip freely. The sample was stored at 4°C for 24 h, then taken out and weighed to calculate the drip loss. Immerse the sealed bag containing LT in a 75°C water bath for about 30 min, then taken out and cooled. Surface moisture was wiped off, and it was reweighed to calculate cooking loss. The determination of cooking loss involved one batch of cooking. The cooked meat samples were trimmed parallel to the muscle fibers and then using a texture analyzer equipped with a strain gauge load cell (C-LM3B; Beijing Tianxiangfei Instrument Equipment) to cut perpendicular to the fiber to measure the shear force.

### Muscle components

#### Muscle conventional fractions

The LT were lyophilized for 48 h, then removed and weighed to calculate the water content. Ether extract (EE) was determined by ether extraction; crude protein (CP) content was measured using the Kjeldahl method; ash was analyzed with a high-temperature electric furnace (SX2-12-12G; Beijing Ying’an Meicheng Scientific Instrument).

#### Muscle amino acids

A hydrolysis tube was charged with 8 mL hydrochloric acid (6 mol/L), and 0.03 g of lyophilized the LT was hydrolyzed at 110°C to convert AAs into free forms. Following 22 h of hydrolysis, the solution was adjusted to a final volume of 50 mL, followed by lyophilization of 1 mL. The lyophilized residue was reconstituted with 1 mL hydrochloric acid (0.02 mol/L), and the supernatant (0.8 mL) was membrane-filtered (0.45 μm) before automatic AA analyzer (LA8080; HITACHI).

### Blood biochemical indicators

Levels of serum glucose (GLU), urea nitrogen (UN), aspartate aminotransferase (AST), alanine aminotransferase (ALT), total protein (TP), albumin (ALB), and globulin (GLOB) were determined using kits (Nanjing Jiancheng Bioengineering Institute). The ELISA kit (Meimian Technology) was used to test levels of growth hormone (GH) and insulin-like growth factor-1 (IGF-1).

### Quantitative real-time polymerase chain reaction

Muscle samples (0.1 g) was added to 1 mL of TRIzol reagent (Takara Biomedical Technology) to extract total RNA. Reverse transcription quantitative polymerase chain reaction (RT-qPCR) was performed using kits from Takara to detect mRNA expression levels. Primers were designed based on the *SUS scrofa genes* published in NCBI. The assay results were calculated using the 2^−ΔΔCt^ method. RT-qPCR primer sequences are provided in Supplement 2.

### Western blotting

Muscle samples (0.1 g) was ground at low temperature in RIPA lysis buffer (Solarbio) containing PMSF (Solarbio Biotechnology) and phosphatase inhibitor (Solarbio). Proteins were separated by electrophoresis on 12% SDS-PAGE gels for S6K1, 15% gels for 4E-BP1, and 6% gels for mTOR. The separated proteins were subsequently transferred to polyvinylidene difluoride (PVDF) membranes (Millipore). Incubation of membrane with diluted primary antibody: GAPDH (ABclonal Technology; 1:40,000), total mTOR, phosphorylated mTOR (Ser2448), total S6K1, total 4E-BP1 (all from Proteintech Group; 1:40,000, 1:5,000, 1:8,000, and 1:4,000, respectively), phosphorylated S6K1 (Thr389) (Abcam; 1:600), and phosphorylated 4E-BP1 (Thr46) (ProMab Biotechnologies; 1:600). The PVDF membrane washed with 1× TBST was incubated with diluted secondary antibody at 37°C for 1 h. Finally, protein bands were visualized using a gel imaging system (Alpha Innotech), and data were quantified with ImageJ software (NIH).

### Statistical analysis

Growth performance data from each stage in this experiment were analyzed using a linear mixed model (SPSS 26.0; IBM). Dietary protein level and weight stage were treated as fixed effects, while individual pig random intercepts served as random effects. Interaction effects were assessed for significance. For interactions found to be significant (p<0.05), simple effects analysis was conducted, and multiple comparisons were performed using the LSD method. Spearman’s correlation analysis was performed on growth performance and meat quality data. All other data were analyzed using one-way analysis of variance. Results were visualized using GraphPad Prism 9 (GraphPad Software). Values are presented as mean± SEM, and statistical significance was set at p<0.05.

## RESULTS

### Growth performance

The results of growth performance (Table 1) showed that the main effect of dietary protein level did not significantly influence ADG (p>0.05). However, the main effects of weight stage and the interaction between protein level and weight stage significantly affected ADG (p<0.05). Specifically: during the 25–50 kg, T4 showed a less ADG compared to the other groups. Conversely, during the 50–75 kg, T4 exhibited a higher ADG. At 100–125 kg, the ADG of T2 was significantly higher than that of T3. The main effect of dietary protein level on ADFI was not significant (p>0.05), but the main effect of weight stage and the interaction between protein level and weight stage significantly influenced ADFI (p<0.05). T4 exhibited significantly lower ADFI than T1 during the 25–50 kg. During the 50–75 kg, the ADFI of T2 was lower than that of the other groups. Both the main effects of dietary protein level and weight stage, as well as their interaction, significantly influenced F/G (p<0.05). During the 25–50 kg, the F/G of T4 was significantly higher than in the other groups. During the 50–75 kg, the F/G of T2 and T4 was significantly lower than that of T1 and T3. During the 100–125 kg, T1 and T2 significantly reduced F/G compared to T3. Significant differences in F/G existed among all dietary treatment groups throughout the entire rearing period (p<0.05). Compared to T3 and T4, T2 significantly reduced F/G, while T1 exhibited a significantly lower F/G than T3.

### Meat quality

**Figure 1:**
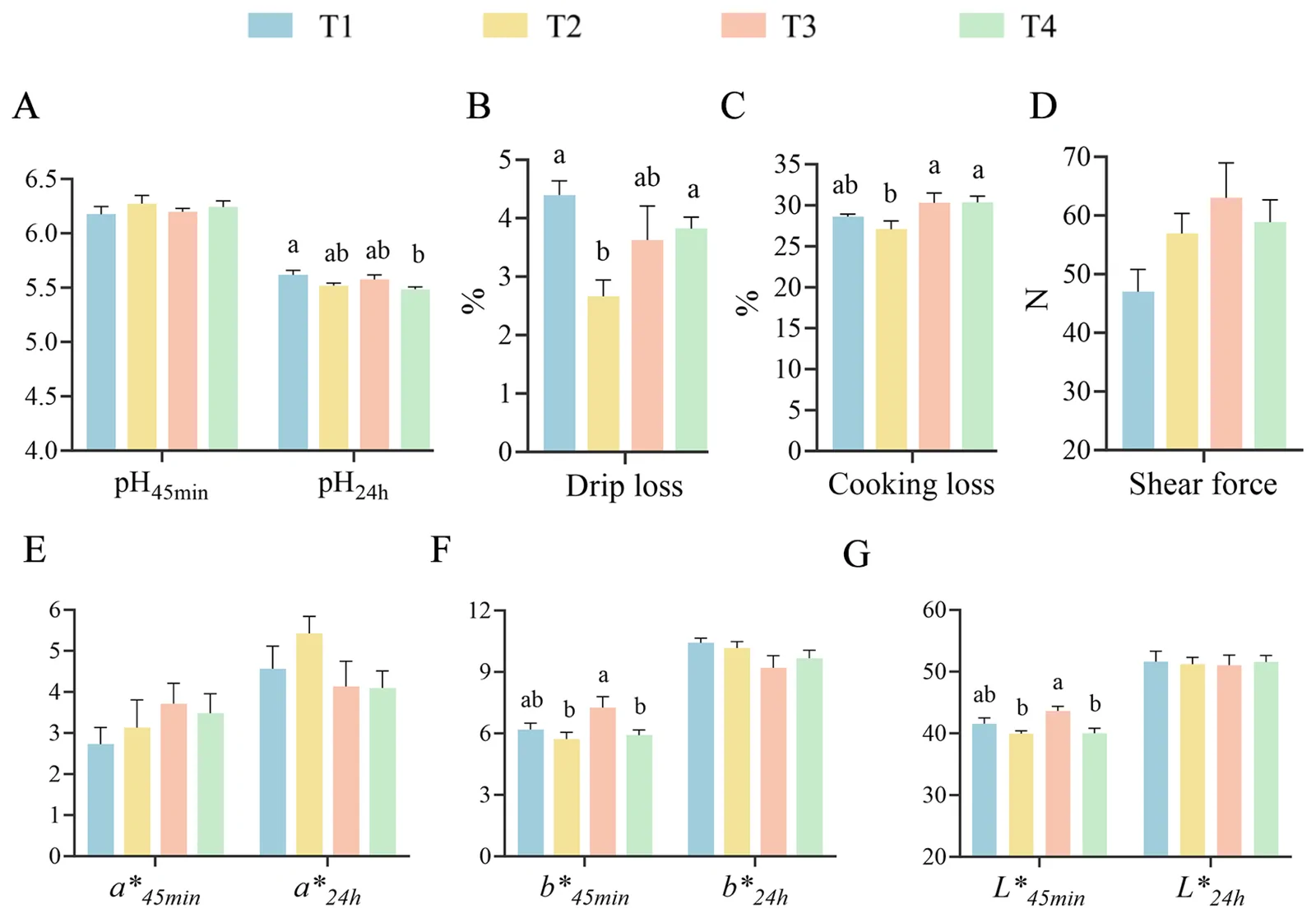
Effects of dietary protein levels on the meat quality of growing-finishing pigs. (A) pH, (B) drip loss, (C) cooking loss, (D) shear force, (E) a* (redness), (F) b* (yellowness), (G) L* (lightness). T1: Dietary protein levels were 15.93%, 14.92%, 13.56% and 11.36% for pigs at 25–50 kg, 50–75 kg, 75–100 kg and 100–125 kg, respectively; T2, T3, and T4: Dietary protein levels at each stage were 104%, 108% and 112% of those in T1, respectively. Values are expressed as mean±standard error. ^a,b^ Different letters indicate significant differences among treatment groups (p<0.05) (n = 6). pH_45min_, postmortem pH at 45 min; pH_24h_, postmortem pH at 24 h; a*_45min_, b*_45min_, L*_45min_, postmortem a*, b*, L* at 45 min; a*_24h_, b*_24h_, L*_24h_, postmortem a*, b*, L* at 24 h.

As illustrated in Figure 1, the pH_24h_ of the LT in T4 was significantly lower than that in T1 (p<0.05). The drip loss of T2 was lower than those of T1 and T4 (p<0.05). Notably, T2 exhibited significantly reduced cooking loss when compared to T3 and T4 (p<0.05). The L*_45min_ and b*_45min_ of the LT in T3 were higher than those in T2 and T4 (p<0.05).

### Nutritional components of meat

**Figure 2:**
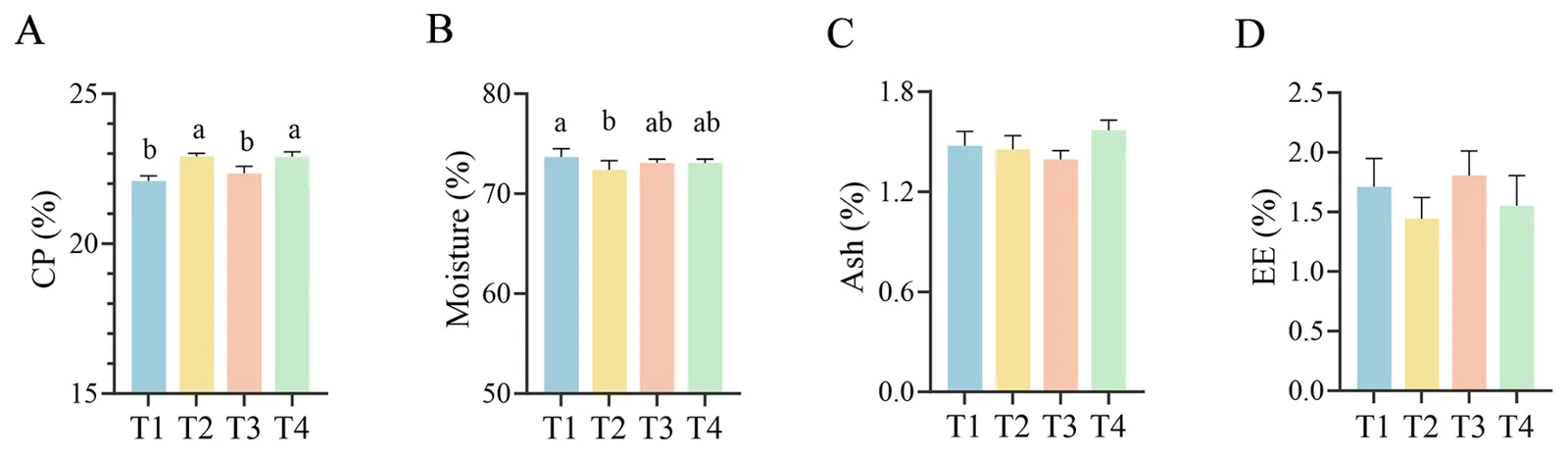
Effects of dietary protein levels on the conventional nutritional composition of the LT muscle. (A) Crude protein (CP), (B) moisture, (C) crude ash, (D) ether extract (EE). T1: Dietary protein levels were 15.93%, 14.92%, 13.56% and 11.36% for pigs at 25–50 kg, 50–75 kg, 75–100 kg and 100–125 kg, respectively; T2, T3, and T4: Dietary protein levels at each stage were 104%, 108% and 112% of those in T1, respectively. Values are expressed as mean±standard error. ^a,b^ Different letters indicate significant differences among treatment groups (p<0.05) (n = 6). LT, longissimus thoracis.

**Figure 3:**
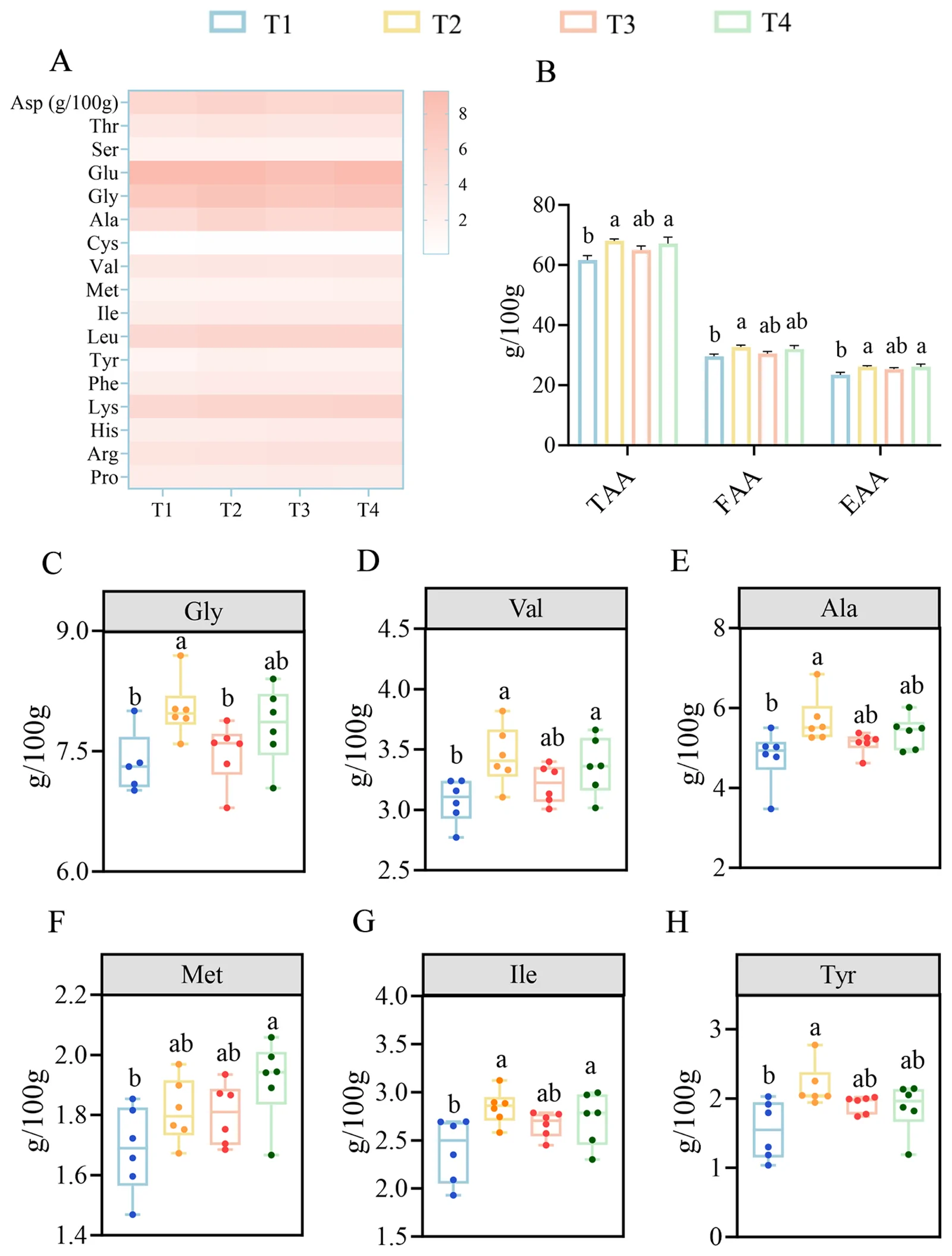
Effects of different protein levels on amino acid concentrations in the LT muscle. (A) Amino acid content. (B) Concentrations of total amino acids, flavor amino acids, and essential amino acids. (C) Glycine, (D) valine, (E) alanine, (F) methionine, (G) isoleucine, and (H) tyrosine levels. T1: Dietary protein levels were 15.93%, 14.92%, 13.56% and 11.36% for pigs at 25–50 kg, 50–75 kg, 75–100 kg and 100–125 kg, respectively; T2, T3, and T4: Dietary protein levels at each stage were 104%, 108% and 112% of those in T1, respectively. Values are expressed as mean±standard error. ^a,b^ Different letters indicate significant differences among treatment groups (p<0.05) (n = 6). LT, longissimus thoracis.

The proximate nutrient composition of the LT in growing-finishing pigs is illustrated in Figure 2. Compared with T1 and T3, CP content in the LT of T2 and T4 were significantly increased, and the moisture in the LT of T2 was significantly lower than that of T1 (p<0.05). Figure 3 illustrates the AA concentration in the LT of growing-finishing pigs. Compared with T1, T2 and T4 showed varying degrees of increased AA concentrations in the muscle. Specifically, the glycine (Gly) content in T2 was elevated compared to T1 and T3, while tyrosine (Tyr), alanine (Ala) and FAA were all significantly higher than T1 (p<0.05). Compared with T1, the methionine (Met) content in T4 was significantly elevated. Additionally, the levels of valine (Val), isoleucine (Ile), total amino acids (TAA) and essential amino acids (EAA) in T2 and T4 were also significantly higher than T1 (p<0.05).

### Blood biochemistry

Table 2 shows the effects of dietary protein levels on blood biochemical in growing-finishing pigs under cold conditions. The UN in T3 was increased compared with T1 (p<0.05). The ALT in T4 was higher than those in the other groups (p<0.05). The ALB and the ALB/GLOB were higher in T2, T3 and T4 than those in T1 (p<0.05).

### Expression of genes involved in protein metabolism and mammalian target of rapamycin pathway protein abundance

**Figure 4:**
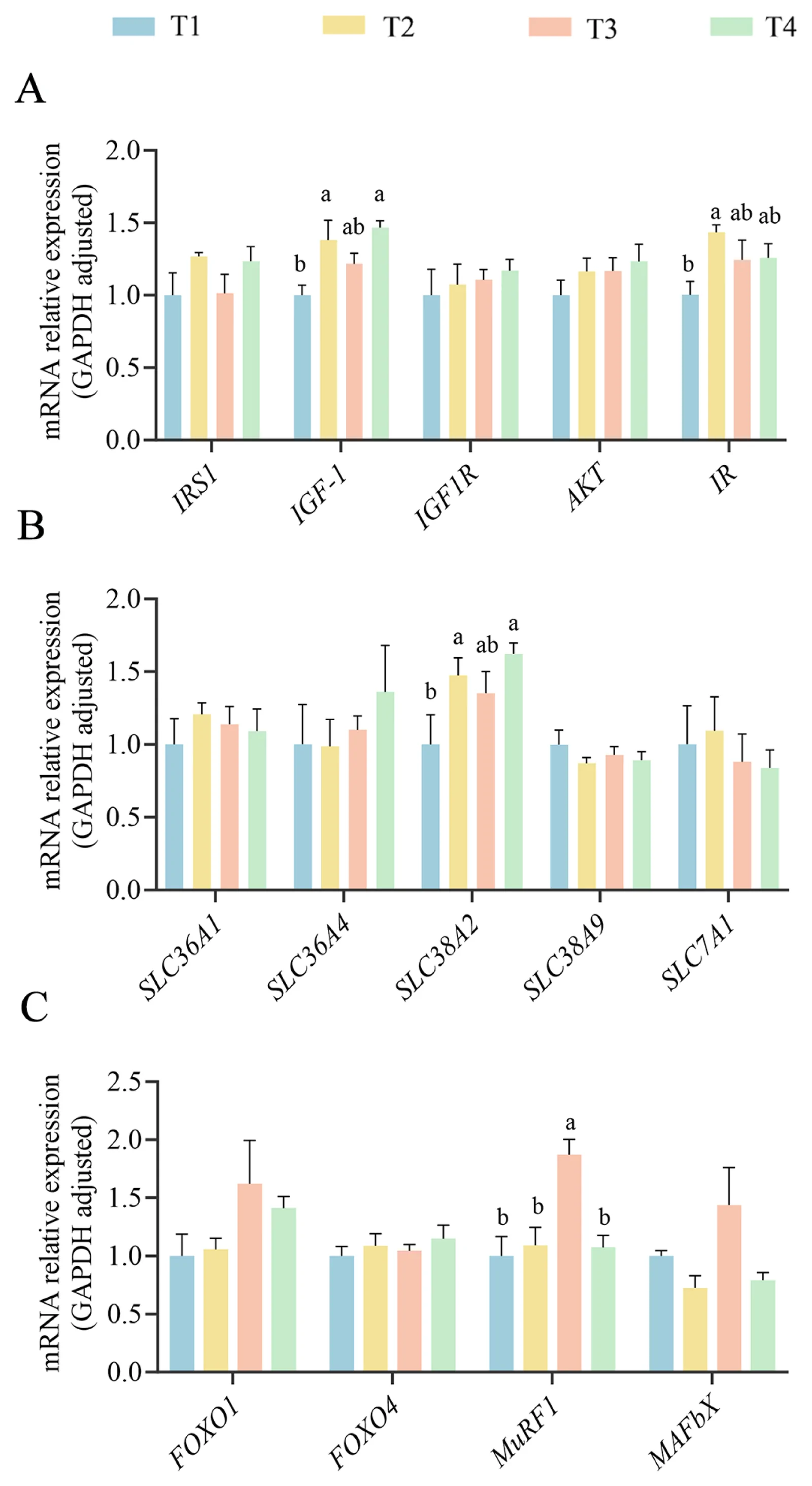
Effects of dietary protein levels on genes related to protein metabolism in the LT muscle (quantitative real-time PCR). (A) Expression levels of protein synthesis-related genes. (B) mRNA expression levels of amino acid transporter proteins. (C) Expression levels of protein degradation-related genes. T1: Dietary protein levels were 15.93%, 14.92%, 13.56% and 11.36% for pigs at 25–50 kg, 50–75 kg, 75–100 kg and 100–125 kg, respectively; T2, T3, and T4: Dietary protein levels at each stage were 104%, 108% and 112% of those in T1, respectively. Values were normalized and are expressed as mean±standard error. ^a,b^ Different letters indicate significant differences among treatment groups (p<0.05) (n = 6). LT, longissimus thoracis; PCR, polymerase chain reaction.

**Figure 5:**
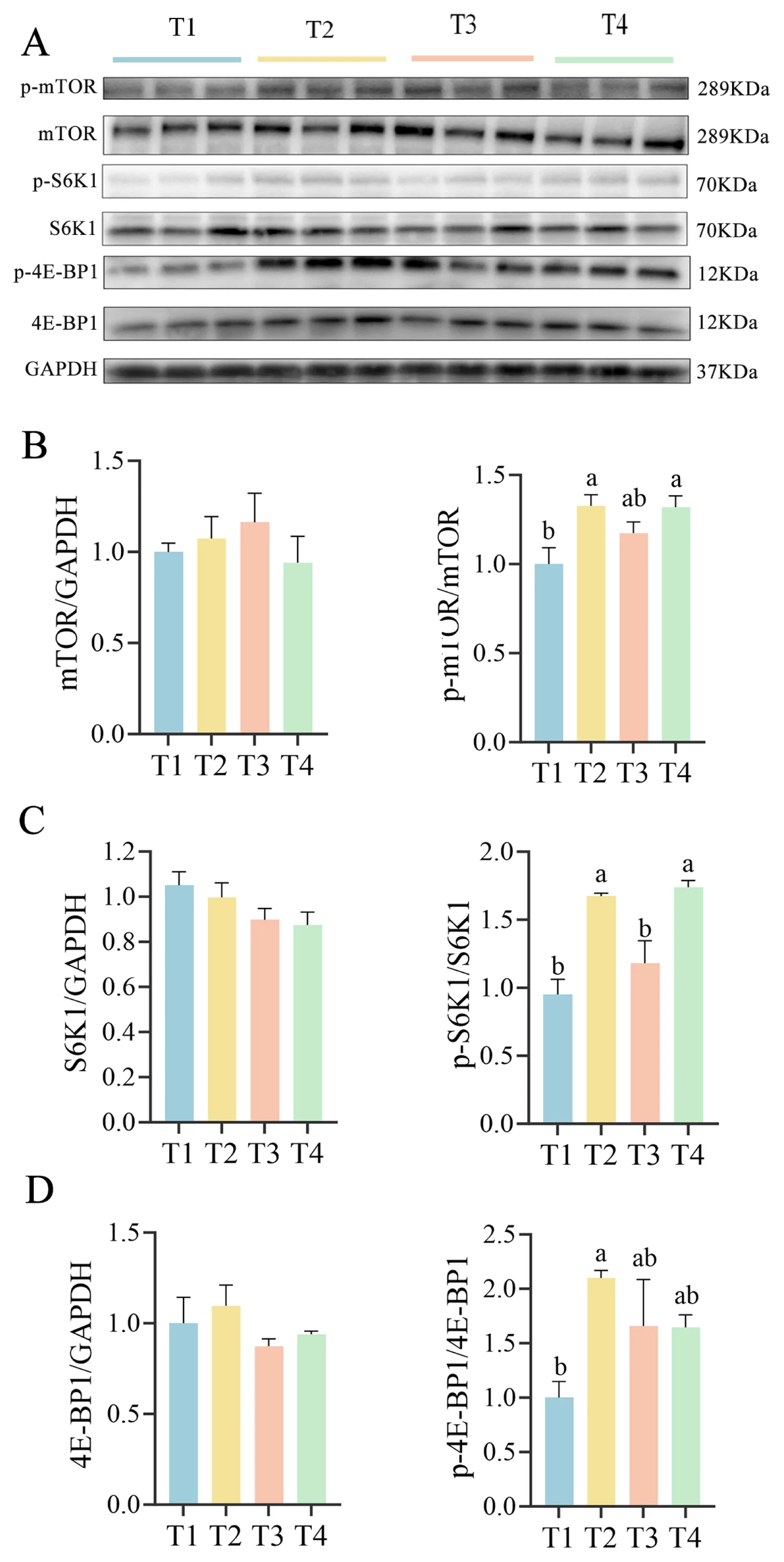
Effects of dietary protein levels on the expression of mTOR signaling pathway-related proteins in the LT muscle. (A) Western blot analysis of the expression and phosphorylation levels of key proteins in the mTOR pathway. (B) Total and phosphorylated mTOR protein expression. (C) Total and phosphorylated S6K1 protein expression. (D) Total and phosphorylated 4E-BP1 protein expression. T1: Dietary protein levels were 15.93%, 14.92%, 13.56% and 11.36% for pigs at 25–50 kg, 50–75 kg, 75–100 kg and 100–125 kg, respectively; T2, T3, and T4: Dietary protein levels at each stage were 104%, 108% and 112% of those in T1, respectively. Values were normalized and are expressed as mean±standard error. ^a,b^ Different letters indicate significant differences among treatment groups (p<0.05) (n = 3). mTOR, mammalian target of rapamycin; S6K1, S6 kinase 1; 4E-BP1, 4E binding protein 1; LT, longissimus thoracis.

As shown in Figure 4, the mRNA expression levels of *IGF-1* in the LT were significantly increased in T2 and T4 compared to T1, and the *IR* expression in T2 was also higher than that in T1 (p<0.05). The mRNA expression levels of *SLC38A2* in T2 and T4 were higher than that in T1 (p<0.05). Compared with other groups, the expression levels of *MuRF1* were significantly increased in T3 (p<0.05). The results in Figure 5 demonstrates that the protein expression of total mTOR, S6K1 or 4E-BP1 were unaffected by dietary protein concentrations during cold environments (p>0.05). However, compared with T1, the p-mTOR/mTOR and p-S6K1/S6K1 were increased in both T2 and T4; moreover, the p-4E-BP1/4E-BP1 was significantly increased in T2 (p<0.05).

### Correlation analysis

**Figure 6:**
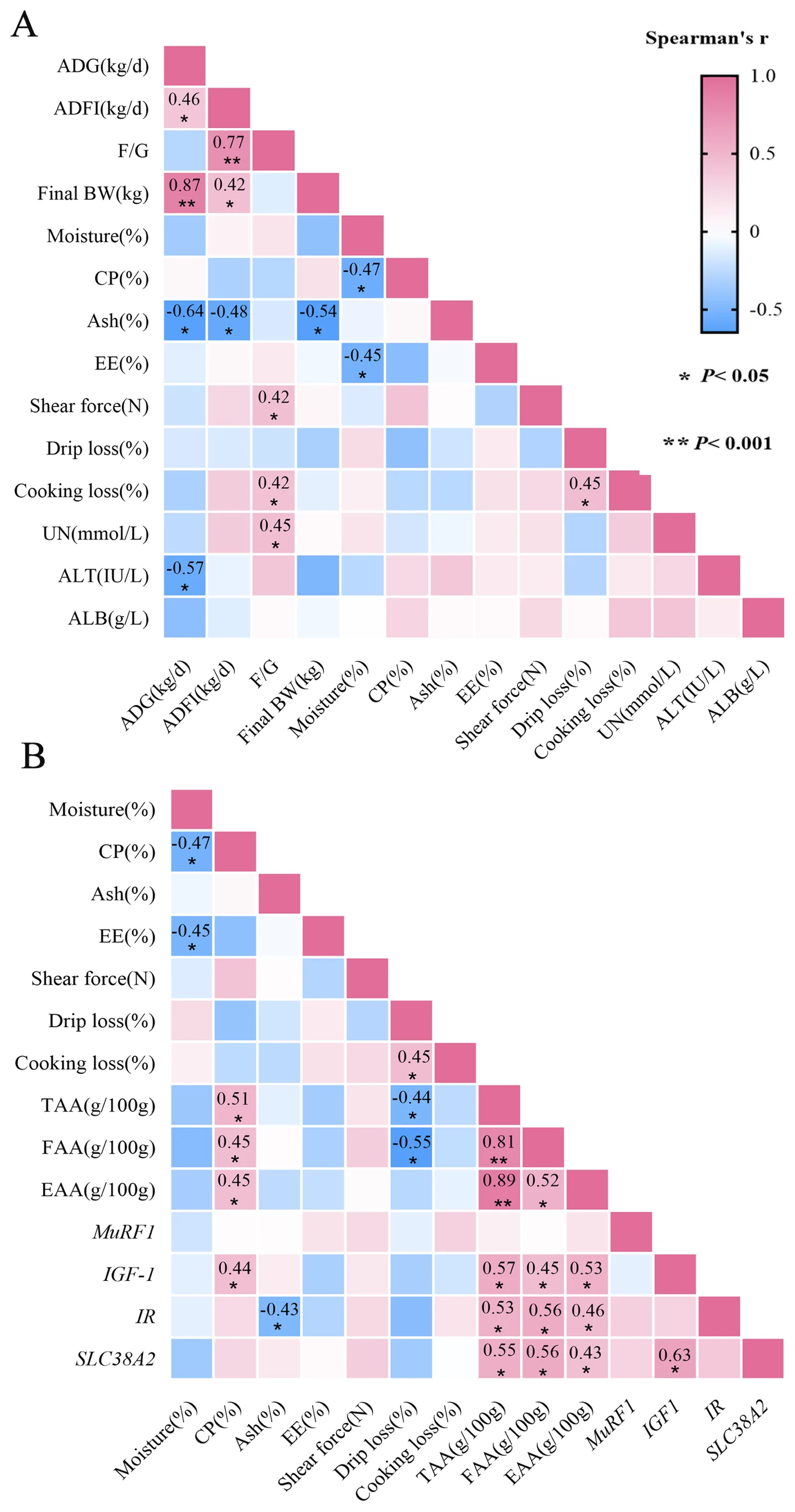
Correlation Analysis. (A) Correlation between growth performance, meat quality and serum biochemical indicators. (B) Correlation between meat quality, nutritional composition and gene expression in protein turnover. * p<0.05; ** p<0.001. ADG, average daily gain; ADFI, average daily feed intake; F/G, feed/gain; CP, protein; EE, ether extract; UN, urea nitrogen; ALT, alanine aminotransferase; ALB, albumin; TAA, total amino acids; FAA, flavor amino acid; EAA, essential amino acids.

There exists a correlation among meat quality-related indicators (Figure 6). Regarding growth performance, ADG showed a positive correlation with ADFI (r = 0.46) and Final BW (r = 0.87). ADFI also exhibited positive correlations with F/G (r = 0.77) and Final BW (r = 0.42). Moisture content was negatively correlated with CP (r = −0.47) and EE (r = −0.45), while ash content was negatively correlated with ADG (r = −0.64) and ADFI (r = −0.48). Among meat quality, shear force positively correlated with F/G (r = 0.42), while cooking loss positively correlated with drip loss (r = 0.45), reflecting muscle water-holding capacity characteristics. Regarding serum biochemical indicators, UN positively correlated with F/G (r = 0.45), ALT negatively correlated with ADG (r = −0.57). Furthermore, muscle nutritional components linked to protein synthesis indicators. CP content positively correlated with TAA (r = 0.51), FAA (r = 0.45), and EAA (r = 0.45), while TAA (r = −0.44) and FAA (r = −0.55) negatively correlated with drip loss. Meanwhile, the mRNA expression levels of *IGF-1*, *IR* and *SLC38A2*, which promote muscle protein synthesis, showed a synergistic upregulation trend, with *IGF-1* significantly positively correlated with muscle CP content (r = 0.44). Additionally, the concentrations of TAA, FAA, and EAA were all positively correlated with the mRNA expression levels of *IGF-1*, *IR*, and *SLC38A2*.

## DISCUSSION

In low-temperature winter environments, pigs’ basal metabolic rates increase and protein breakdown intensifies; therefore, adequate protein intake is essential for maintaining growth, development, and productivity. The results of this study showed that, throughout the experimental period, the F/G was significantly lower in T2 compared to T3 and T4, suggesting that excessively high dietary protein levels may increase production costs due to protein wastage. Pigs weighing 25–50 kg are in the growth phase, characterized by rapid muscle and bone development and high protein requirements [9]. However, due to the immature digestive system of pigs at this stage, excessive protein intake will increase intestinal burden, thereby inhibiting ADFI, reducing ADG and elevating F/G [10], which compromises farmer profitability. Similarly, our research found that during this stage, T4 had lower ADFI and ADG, but an increased in F/G, which may be due to insufficient energy intake caused by low ADFI, resulting in slow growth during this stage [11]. As pig body weight increases, the absorption and utilization efficiency of nutrients in the diet also rises [12]. In this study, the ADG of T4 was higher than that of other groups during the 50–75 kg stage, alongside a lower F/G compared to T1 and T3. Therefore, providing a higher dietary protein level during this stage may promote rapid growth in pigs. This may be attributed to the progressive maturation of the digestive system during this stage, allowing greater protein absorption and utilization, thereby reducing the feeding days in this stage. Although the weight gain rate of T4 accelerated at this stage, it still cannot fully compensate for the growth retardation caused by insufficient energy intake in the early stage. The ADFI of T2 during this phase was lower than that of the other three groups, and F/G was also lower than that of T1 and T3. This suggests that T2’s digestibility may have improved [13], sufficient to meet maintenance requirements under low-temperature conditions. During the late finishing phase (100–125 kg), pigs primarily accumulate fat, reducing protein requirements. The ADG of T2 was significantly higher than that of T3 and showed an upward trend compared to T1. This growth advantage was likely the primary reason for the shorter rearing period in T2 compared to the other groups. Analysis of the various growth stages indicated that, although there were no significant differences in overall daily weight gain among the treatment groups, the growth advantage exhibited by T2 at each stage resulted in a shorter total rearing cycle, which has positive implications for reducing labor, management, and heating costs. Furthermore, the F/G of T1 and T2 was lower than that of T3, indicating that appropriately reducing dietary protein levels during this phase not only meets the growth requirements of finishing pigs but also reduces F/G, thereby controlling production costs. Low temperatures increase the maintenance requirements of pigs and elevate their energy needs. Research found that raising the net energy level for growing-finishing pigs by 6% above the NRS recommendation could mitigate the negative effects of low temperatures on pig growth [8]. Building on this, the present study investigated the optimal dietary protein level for growing-finishing pigs under low-temperature conditions and found that under a uniformly elevated net energy level, moderately increasing the protein level improved growth performance and shortened the production cycle. This suggests that the optimal energy-to-protein ratio for pigs under low-temperature may differ from that under normal temperature.

Meat quality as an important indicator of pork consumption quality, evaluated by pH, color, cooking loss, drip loss, and tenderness, influences consumer choices. Previous studies have indicated that under suitable temperature conditions, dietary protein levels have no effect on the pH value of pig muscle [14], but our study found that in low-temperature conditions, the pH_24h_ of LT in T4 was lower than that in T1, which may negatively impact meat tenderness [15]. Furthermore, this study found that compared to T3, the L* and b* of the LT were reduced in T2 and T4 at 45 min postmortem, and consumers typically prefer meat products with higher a* and lower b* [16], given that no color differences were observed among the groups after 24 h, it is inferred that their impact on actual consumer preferences is likely limited. Higher drip loss indicates poorer sensory quality in meat (such as poor tenderness, flavor intensity, etc.) [17]. In this investigation, T2 had lower drip loss than T1 and T4, we speculate that this may be due to T2 having a more stable muscle cytoskeletal structure, which reduces myofibrillar contraction and retains more water within the muscle [18]. Concurrently, we observed a positive correlation between drip loss and cooking loss in LT, which may also explain the lower cooking loss in T2. These results may indicate that pork from T2 exhibits higher marbling [19]. Moreover, increased F/G correlate with increased shear force and greater cooking losses in pork, resulting in diminished tenderness and water-holding capacity of meat products. This indicates that feed conversion efficiency impacts not only economic returns but also meat quality. Consequently, winter farming should consider the interplay between growth performance and meat quality, aiming to improve pork quality and consumer appeal while optimizing cost efficiency.

With growing health awareness, conventional meat products have become inadequate to satisfy contemporary consumer requirements, they prioritize meat that is nutritionally dense, flavorful, and has a higher energy density, typically featuring elevated levels of protein and AA [20]. Dietary protein typically does not affect muscle moisture [14], but in this study, T2 had lower moisture than T1, possibly indicating increased marbling [21], and enhanced palatability. Animal muscle is a high-quality protein that provides a richer source of nutrients than plant-source proteins. Low temperatures decrease muscle protein synthesis and meat nutritional value, while dietary protein correlates positively with muscle protein deposition [22]. Balanced digestible AA supply enhances AA utilization [23], whereas imbalance may promote protein degradation. In this study, T2 and T4 had higher muscle CP than T1, but T3 showed no difference, possibly due to increased muscle protein degradation. Furthermore, we observed that moisture in LT exhibited a negative correlation with CP and EE. This may be attributable to CP and EE replacing moisture deposits within the tissue [24]. Skeletal muscle is the most significant AA pool in the animals. AAs are not only key factors in determining the quality of pork but also an important basis for assessing its nutritional value [25,26]. More importantly, AAs as flavor precursors that can undergo chemical reactions to produce volatile aromatic compounds, which are key to forming the enticing flavor of meat. Meat products that are both highly nutritious and flavorful are precisely what contemporary consumers seek. Low temperatures reduce Val, Leu, and Ile in Yorkshire pig muscle [27], while increased dietary protein raises the concentration of most AAs in pig muscles [28]. Our study showed that T2 and T4 increased AA concentrations in the LT, with T2 showing increased levels of total TAA, EAA, and FAA, likely from reduced AA limitation after moderate protein intake [29]. This study found that the content of TAA and FAA in LT was negatively correlated with drip loss. This may be because AA help maintain myofibrillar protein structure, thereby improving the water-holding capacity of muscle [30]. Consequently, increasing dietary protein can mitigate nutrient loss caused by low temperatures, enhance muscle protein and AA content. Since high-protein foods can meet nutritional needs with smaller portions, consumers are increasingly inclined to choose high-protein products and are willing to pay a premium [31]. Therefore, raising the protein content in pork products helps enhance their appeal to consumers, thereby improving their market competitiveness.

Dietary protein levels significantly influence serum biochemical parameters in pigs. UN reflects protein catabolism: higher UN indicates lower protein utilization, and high-protein diets increase UN. In this experiment, we found that the UN content of T3 was higher than that of T1, which indicated that protein decomposition in the body was accelerated. Interestingly, the UN content of T4 did not show significant difference, which we hypothesized might be due to an imbalance in the ratio of digestible AAs in T3’diet, leading to increased AA deamination and consequently elevated UN content [32]. Correlation analysis indicates a positive relationship between UN and F/G, suggesting that growing-finishing pigs with low feed utilization may exhibit higher protein breakdown, leading to nutrient wastage. Blood ALB and GLOB reflect nutritional and immune status, with a high ALB/GLOB being beneficial [33]. Increasing dietary protein raises ALB/GLOB in pigs [10]. Similarly, our findings showed that T2, T3, and T4 had higher ALB and ALB/GLOB than T1, indicating improved nutritional status under cold conditions. ALT and AST reflect liver health, one characteristic of liver damage is the massive release of AST and ALT. In this experiment, T4 had higher ALT than other groups, but no significant alterations in AST activity were detected, suggesting liver metabolic burden and mild damage. It is noteworthy that serum ALT levels were negatively correlated with ADG. We speculate that the growth retardation observed in T4 may be attributable to the metabolic burden caused by excessively high dietary protein levels. Thus, exceeding T3’s protein level may harm animal health.

Animal protein metabolism balances synthesis and degradation via mTOR-mediated synthesis and ubiquitin-proteasome-mediated degradation. IGF-1, an mTOR upstream regulator, promotes protein synthesis by activating IRS-1. This study found that the mRNA expression levels of *IGF-1* were upregulated in both T2 and T4, consistent with the existing knowledge that protein levels influence *IGF-1* expression [34]. Similarly, IR binding to insulin activates mTOR by phosphorylating IRS [35]. *IR* expression in the LT of T2 was significantly higher than in T1, indicating that T2 more effectively activates the mTOR pathway to promote muscle CP deposition. Analysis of phosphorylation levels at key signaling nodes provided evidence for these regulatory mechanisms. T2 and T4 enhanced p-mTOR and its downstream effector p-S6K1 compared with T1, driving translation initiation and ultimately leading to increased CP and AA content in the muscles of T2 and T4. Given that 4E-BP1 plays a critical role in releasing translation initiation inhibition, it is more important than S6K1 in protein synthesis [36]. Consequently, increased p-4E-BP1 levels in T2 likely conferred the strongest protein deposition capacity to this group. This may account for the production of premium meat products with lower moisture content and higher nutritional value in T2. we hypothesize that the lower F/G observed in this group may reflect its ability to convert feed more efficiently into high-quality lean meat. The primary function of MuRF1 in skeletal muscle is to inhibit protein synthesis and promote degradation. T3 had higher *MuRF1* expression, possibly due to digestible AA imbalance [37], explaining its unchanged muscle protein. AA serve as a nutritional signal to activate mTOR. In this study, AA transporter SLC38A2 mediates branched-chain AA uptake and exhibits upregulation in both T2 and T4. This finding aligns with prior research indicating that elevated dietary protein levels enhance *SLC38A2* expression, thereby increasing cellular AA transport capacity and subsequently activating mTOR via Rag GTPase [38]. Notably, *IGF-1*, *IR*, and *SLC38A2* exhibited synergistic upregulation trends, this suggests that appropriately increasing dietary protein may promote efficient protein deposition by synergistically activating the mTOR pathway through upregulating protein synthesis-related genes and enhancing cellular nutrient uptake capacity. We further analyzed the association between muscle AA and CP synthesis-related genes. The results showed that the concentrations of TAA, FAA, and EAA in muscle exhibited varying degrees of positive correlation with the mRNA expression levels of *IGF-1*, *IR*, and *SLC38A2*. This suggests that AA deposited within LT may themselves act as a nutritional signal, positively regulating the IGF-1/insulin signaling pathway [39]. Collectively, these findings show that appropriate protein supplementation under cold conditions enhances muscle protein synthesis via the mTOR pathway, improving meat nutritional value and encouraging consumer purchases. Therefore, protein supply levels must be adjusted according to environmental factors in production practice to better meet consumer demand for nutrient-dense, high-quality meat products.

In view of the continuous shortage and rising prices of protein feed resources, simply increasing dietary protein supply is not a cost-effective feeding strategy, and any adjustment of dietary protein content should be comprehensively evaluated based on input costs and economic benefits. Because pigs’ protein requirements change at low temperatures, determining the appropriate protein intake while ensuring production performance is beneficial for promoting swine production in cold regions. The present study focused on exploring the appropriate dietary protein level for growing-finishing pigs exposed to low ambient temperatures. In general, a higher dietary protein level will inevitably lead to increased feed input costs across different growth stages. Nevertheless, moderate protein supplementation under low-temperature conditions still presents favorable economic benefits and practical application value. Appropriately elevated dietary protein can effectively shorten the overall feeding cycle of finishing pigs. Cold seasons require continuous barn temperature maintenance, which brings sustained high fixed expenditures including heating, labor, facility operation and maintenance, electricity, and site occupation. A shortened rearing period can greatly reduce such daily comprehensive management and heating costs, and also decrease the total feed consumption throughout the production cycle. Moreover, reasonable protein improvement can optimize meat quality by reducing drip loss and promoting the deposition of CP and AAs in muscle tissue. With the continuous upgrading of consumer demand, high-quality meat products with superior nutritional properties can further improve market acceptance and consumer preference. Collectively, moderately increasing dietary protein to a proper proportion of the standard recommendation is a feasible nutritional regulation strategy under low-temperature environments, which can comprehensively balance growth performance, production cost control, and meat quality improvement.

## CONCLUSION

The study showed that increasing dietary protein content to 104% of the NRS recommended level under winter low-temperature conditions serves as a viable nutritional strategy to shorten production cycles and enhance pork quality. Furthermore, this approach significantly increased AA and protein content in pork by activating the mTOR/S6K1/4E-BP1 pathway, thereby optimizing flavor characteristics and overall nutritional quality. These findings underscore the value of optimizing protein supplementation strategies under low-temperature farming conditions, providing reference for the production of high-protein pork products.

## Figures and Tables

**Table 1:** Effect of dietary protein levels on growth performance of growing-finishing pigs under low temperature environment

Items	Treatments				p-value 11)	p-value 22)	p-value 33)

	T1	T2	T3	T4			
ADG (kg/d)							
25–50 kg	0.80±0.03^a^	0.76±0.03^a^	0.78±0.03^a^	0.50±0.04^b^	<0.001	0.409	<0.001
50–75 kg	0.98±0.03^b^	0.97±0.03^b^	0.91±0.03^b^	1.22±0.04^a^			
75–100 kg	1.04±0.04	1.10±0.04	1.11±0.04	1.11±0.04			
100–125 kg	0.91±0.04^ab^	1.01±0.04^a^	0.90±0.04^b^	1.00±0.05^ab^			
ADFI (kg/d)							
25–50 kg	1.71±0.06^a^	1.55±0.06^ab^	1.54±0.06^ab^	1.46±0.07^b^	<0.001	0.206	<0.001
50–75 kg	2.42±0.09^a^	2.06±0.09^b^	2.48±0.09^a^	2.64±0.11^a^			
75–100 kg	3.08±0.13	3.20±0.13	3.39±0.13	3.20±0.16			
100–125 kg	3.12±0.13	3.43±0.13	3.37±0.13	3.42±0.15			
F/G							
25–50 kg	2.14±0.08^b^	2.05±0.08^b^	2.01±0.09^b^	2.80±0.10^a^	<0.001	<0.001	<0.001
50–75 kg	2.47±0.06^b^	2.14±0.06^c^	2.74±0.06^a^	2.17±0.07^c^			
75–100 kg	2.94±0.08	2.93±0.08	3.07±0.08	2.89±0.09			
100–125 kg	3.47±0.13^b^	3.40±0.13^b^	3.87±0.14^a^	3.45±0.16^ab^			
The whole trial period							
ADG (kg/d)	0.96±0.01	0.97±0.09	0.95±0.01	0.90±0.03	-	0.125	-
ADFI (kg/d)	2.58±0.07	2.52±0.08	2.72±0.08	2.53±0.09	-	0.295	-
F/G	2.68±0.04^bc^	2.60±0.04^c^	2.87±0.06^a^	2.79±0.04^ab^	-	0.001	-
Final BW (kg)	129.63±2.06	132.81±2.75	130.38±1.74	125.24±3.19	-	0.228	-
Feeding days (d)					-	-	-
25–50 kg	31	31	30	46	-	-	-
50–75 kg	26	27	28	20	-	-	-
75–100 kg	23	21	22	21	-	-	-
100–125 kg	27	24	27	24	-	-	-
The whole trial period	107	103	107	111	-	-	-

The values are the mean±standard error.

T1: Dietary protein levels were 15.93%, 14.92%, 13.56% and 11.36% for pigs at 25–50 kg, 50–75 kg, 75–100 kg and 100–125 kg, respectively; T2, T3, and T4: Dietary protein levels at each stage were 104%, 108% and 112% of those in T1, respectively.

1) p-value (stage).

2) p-value (protein)

3) p-value (protein×stage).

a–c Different letters indicate significant differences among treatment groups (p<0.05) (n = 12).

ADG, average daily gain; ADFI, average daily feed intake; F/G, feed/gain.

**Table 2:** Effect of dietary protein levels on growth performance of pigs under low temperature environment

Items	Treatments				p-value

	T1	T2	T3	T4	
UN (mmol/L)	3.46±0.16^b^	3.85±0.35^ab^	5.16±0.18^a^	4.29±0.38^ab^	0.001
GLU (mmol/L)	5.54±0.15	4.63±0.43	5.86±0.23	5.66±0.16	0.179
ALT (IU/L)	53.32±3.12^b^	60.60±3.96^b^	61.28±4.54^b^	73.56±3.39^a^	0.010
AST (IU/L)	25.92±1.70	32.52±3.38	29.25±3.09	23.42±3.92	0.236
TP (g/L)	66.12±4.30	71.24±1.54	75.57±2.14	70.64±1.35	0.256
ALB (g/L)	37.97±2.76^b^	44.93±1.23^a^	47.97±1.67^a^	46.02±2.02^a^	0.012
GLOB (g/L)	31.07±2.82	26.31±1.41	27.60±1.63	24.62±0.38	0.115
ALB/GLOB	1.26±0.12^b^	1.74±0.11^a^	1.78±0.13^a^	1.87±0.10^a^	0.005
GH (μg/L)	9.95±0.64	10.93±0.98	11.16±0.97	12.01±0.63	0.387
IGF-1 (μg/L)	92.84±4.84	102.71±3.40	92.83±4.71	100.01±3.14	0.240

The values are the mean±standard error.

T1: Dietary protein levels were 15.93%, 14.92%, 13.56% and 11.36% for pigs at 25–50 kg, 50–75 kg, 75–100 kg and 100–125 kg, respectively; T2, T3, and T4: Dietary protein levels at each stage were 104%, 108% and 112% of those in T1, respectively.

a,b Different letters indicate significant differences among treatment groups (p<0.05) (n = 6).

UN, urea nitrogen; GLU, glucose; ALT, alanine aminotransferase; AST, aspartate aminotransferase; TP, total protein; ALB, albumin; GLOB, globulin; GH, growth hormone; IGF-1, insulin-like growth factor-1.

## SUPPLEMENTARY MATERIAL

**Supplement 1.** Composition and nutrient levels of diets.

**Supplement 2.** Primer sequences used for RT-qPCR analysis.
