# Supplementary material for: Dietary protein levels impact growth, meat quality, and muscle protein pathways in growing-finishing pigs under low-temperature conditions
Source: Anim Biosci. 2026 Jun 1;39(9):260002. doi: 10.5713/ab.260002 (PMC13547615; doi:10.5713/ab.260002)
Supplement: Supplementary file 1 [file ab-260002-Supplementary-1.pdf]

Supplement 1. Composition and nutrient levels of diets (air-dried basis, %)

Item	25-50 kg				50-75 kg				75-100 kg				100-125 kg			
	T1	T2	T3	T4	T1	T2	T3	T4	T1	T2	T3	T4	T1	T2	T3	T4
Ingredients																
Corn	70.33	68.33	66.48	64.46	74.62	72.67	70.83	68.88	80.25	78.52	76.85	75.07	81.53	80.63	79.61	77.70
Soybean meal	18.13	20.13	23.07	25.25	18.97	20.70	22.44	24.14	14.60	16.16	17.69	19.32	6.57	8.01	9.38	10.63
Full-fat soybean	4.20	3.98	2.54	2.10	-	-	-	-	-	-	-	-	-	-	-	-
Rice bran	-	-	-	-	-	-	-	-	-	-	-	-	7.52	6.84	6.40	6.87
Soybean oil	3.54	3.78	4.15	4.43	3.13	3.35	3.52	3.75	2.00	2.20	2.36	2.55	1.12	1.27	1.38	1.58
Calcium hydrogen phosphate	0.95	0.90	0.90	0.90	0.71	0.71	0.65	0.65	0.50	0.48	0.48	0.48	0.35	0.35	0.35	0.35
Limestone	0.92	0.95	0.92	0.92	0.99	0.99	1.00	1.00	1.10	1.10	1.07	1.07	1.18	1.18	1.17	1.16
Salt	0.27	0.27	0.27	0.27	0.22	0.22	0.22	0.22	0.23	0.23	0.23	0.23	0.23	0.23	0.23	0.23
L-Lysine	0.43	0.43	0.43	0.43	0.28	0.27	0.26	0.27	0.26	0.26	0.25	0.23	0.37	0.36	0.35	0.35
DL-Methionine	0.07	0.07	0.08	0.08	0.01	0.02	0.02	0.03	-	-	0.01	-	0.01	0.01	0.01	0.01
L-Threonine	0.13	0.13	0.14	0.14	0.06	0.06	0.06	0.06	0.05	0.05	0.06	0.05	0.09	0.10	0.09	0.10
L-Tryptophan	0.03	0.03	0.02	0.02	0.01	0.01	-	-	0.01	-	-	-	0.03	0.02	0.03	0.02
Premix [1)]	1.00	1.00	1.00	1.00	1.00	1.00	1.00	1.00	1.00	1.00	1.00	1.00	1.00	1.00	1.00	1.00

Item	25-50 kg				50-75 kg				75-100 kg				100-125 kg			
	T1	T2	T3	T4	T1	T2	T3	T4	T1	T2	T3	T4	T1	T2	T3	T4
Nutrient levels [2)]																
Digested energy, MJ/kg	14.71	14.75	14.79	14.83	14.54	14.59	14.64	14.69	14.31	14.36	14.40	14.45	13.98	14.03	14.06	14.10
Metabolizable energy, MJ/kg	13.99	14.01	14.02	14.04	13.89	13.91	13.93	13.95	13.74	13.76	13.77	13.79	13.49	13.52	13.54	13.55
Net energy, MJ/kg	10.99	10.99	10.99	10.99	10.92	10.92	10.92	10.92	10.82	10.82	10.82	10.82	10.70	10.70	10.70	10.70
Crude protein	15.93	16.50	17.22	18.05	14.92	15.70	16.32	16.83	13.56	14.15	14.50	15.12	11.36	11.77	12.15	12.72
Crude fat	7.86	7.19	6.72	7.45	6.56	6.20	6.03	5.84	4.65	5.11	4.58	5.10	5.69	5.46	6.46	5.74
Crude fiber	2.79	2.84	2.89	2.94	2.74	2.79	2.84	2.89	2.63	2.68	2.72	2.77	2.68	2.71	2.73	2.75
Lysine	0.97	1.01	1.05	1.09	0.81	0.84	0.87	0.91	0.70	0.73	0.76	0.78	0.60	0.62	0.65	0.67
Methionine	0.29	0.30	0.31	0.32	0.23	0.24	0.25	0.26	0.20	0.21	0.22	0.22	0.17	0.18	0.18	0.19
Threonine	0.60	0.62	0.65	0.67	0.51	0.53	0.55	0.57	0.45	0.47	0.49	0.50	0.38	0.40	0.41	0.43
Tryptophan	0.17	0.18	0.18	0.19	0.14	0.15	0.15	0.16	0.12	0.12	0.13	0.13	0.10	0.10	0.11	0.11
Calcium	0.71	0.65	0.66	0.70	0.62	0.60	0.55	0.59	0.60	0.60	0.58	0.55	0.57	0.56	0.59	0.55
Total phosphorus	0.47	0.46	0.46	0.46	0.42	0.44	0.44	0.45	0.38	0.39	0.36	0.38	0.51	0.45	0.48	0.48
Sodium	0.12	0.12	0.12	0.12	0.10	0.10	0.10	0.10	0.10	0.10	0.10	0.10	0.11	0.11	0.11	0.11
Chlorine	0.20	0.20	0.20	0.20	0.17	0.17	0.17	0.17	0.18	0.18	0.18	0.18	0.18	0.18	0.18	0.18

Note: [1)] Provided per kg diet(25-50 kg): vitamin A(VA), 13,500 IU; vitamin D3 (VD), 3,000 IU; vitamin E (VE), 24 IU; vitamin K3 (VK3), 3 mg;

vitamin B (VB), 13.52 mg; folic acid, 1.5 mg; D-biotin, 0.12 mg; niacin, 30 mg; pantothenic acid, 15 mg; Fe, 70 mg; Cu, 4.5 mg; Mn, 3 mg; Zn, 70 mg; I, 0.14 mg; Se, 0.3 mg. (50-75 kg): VA, 13,500 IU;VD3, 3,000 IU; VE, 24 IU; VK3, 3 mg; VB, 13.52 mg; folic acid, 1.5 mg; D-biotin, 0.12 mg; niacin, 30 mg; pantothenic acid, 15 mg; Fe, 60 mg; Cu, 4 mg; Mn, 2 mg; Zn, 60 mg; I, 0.14 mg; Se, 0.25 mg. (75-100 kg): VA, 13,500 IU; VD3, 3,000 IU; VE, 24 IU; VK3, 3 mg; VB, 13.52 mg; folic acid, 1.5 mg; D-biotin, 0.12 mg; niacin, 30 mg; pantothenic acid, 15 mg; Fe, 50 mg; Cu, 3.5 mg; Mn, 2 mg; Zn, 50 mg; I, 0.14 mg; Se, 0.25 mg. (100-125 kg): VA, 13,500 IU; VD3, 3,000 IU; VE, 24 IU; VK, 3 mg; VB, 13.52 mg; folic acid, 1.5 mg; D-biotin, 0.12 mg; niacin, 30 mg; pantothenic acid, 15 mg; Fe, 40 mg; Cu, 3 mg; Mn, 2 mg; Zn, 50 mg; I, 0.14 mg; Se, 0.2 mg.

[2)] Crude protein, crude fat, calcium and total phosphorus were analyzed values. Other values were obtained through calculation.
