# Supplementary material for: Dietary protein levels impact growth, meat quality, and muscle protein pathways in growing-finishing pigs under low-temperature conditions
Source: Anim Biosci. 2026 Jun 1;39(9):260002. doi: 10.5713/ab.260002 (PMC13547615; doi:10.5713/ab.260002)
Supplement: Supplementary file 2 [file ab-260002-Supplementary-2.pdf]

Supplement 2. Primer sequences used for RT-qPCR analysis (F: forward; R: reverse)

Gene Name	Gen BanK	Primer sequence (5'-3')	Product size (bp)
IGF-1	NM_214256.1	F[1]: CCTGTGCTTGCTCTCCTTCA<br>R[2]: TACCCTGTGGGCTTGTTGAAA	129
IGF-1R	NM_214172.1	F: ATGGAGGAAGTGACAGGGACTA<br>R: GTGGTGGTGGAGGTGAAGTG	116
AKT	NM_001159776.1	F: TCAAGAACGACGGCACCTTCATC<br>R: CGCCACGGAGAAGTTGTTGAGG	89
IRS1	NM_001244489.1	F: CCCTACTATTTCCCACCAGAAG<br>R: CATTTCCAGACCCTCCTCAG	175
IR	NM_214172.1	F: CATACCTGAACGCCAAGAAGTT<br>R: GTCATTCCAAAGTCTCCGATTT	100
FOXO4	XM_003135172.4	F: CCCTCTCATTCTGTCACCTTG<br>R: ATGTGCTCTCCCACCATAGC	103
FOXO1	NM_214014.3	F: CGGCAGGCTGGAAGAATTCAA<br>R: CTCCCTCTGGGTTGAGCATC	114
MAFBX	NM_001044588.1	F: GCTGGATTGGAAGAAGATGTAT<br>R: AGGAAAGAATGTGGCAGTGTCT	104
MuRF1	NM_001184756.1	F: GTCTTCCAGGGACAGAAGACTG<br>R: AGTTTGCATGCGGTCATTCC	75
SLC36A1	XM_021077073.1	F: AAGGCAACATTGGCACAGGACTC<br>R: GGCACACTTCACCAGCAGACC	134
SLC36A4	XM_013979268.2	F: CCCTGATCCTACCGCCCTTGG<br>R: GAAACCAACAACCCCAGTGAATGC	110
SLC38A2	NM_001317081.1	F: GCTTGCTGCCCTCTTTGGATACC<br>R: ACAGCCAAACGGACAATGAGAAGG	117
SLC38A9	XM_005672453.3	F: CCAATGACACGGGACTCCAACAG<br>R: GAAATTGAGCAGTGGCAGGAGGAG	98
SLC7A1	NM_001012613.1	F: GGTCCTGGGCTTCATAATGGTGTC<br>R: TTCGTGGCATTGTTCAGGCAGAG	117
GAPDH	NM_001206359.1	F: AACATCATCCCTGCTTCTACCG<br>R: CAGGTCAGATCCACAACCGACA	128

[1] F, Forward sequence.

[2] R, Reverse sequence.

IGF-1, Insulin-like Growth Factor 1; *AKT*, Protein Kinase B; *IR*, Insulin Receptor; *FOXO4*, Forkhead Box Protein O4; *IGF-1R*, Insulin-like Growth Factor 1 Receptor; *FOXO1*, Forkhead Box Protein O1; *IRS1*, Insulin Receptor Substrate 1; *MAFBX*, Muscle Atrophy F-box Protein; *MuRF1*, Muscle RING Finger Protein 1; *SLC36A1*, Proton-coupled Amino Acid Transporter 1; *SLC36A4*, Proton-coupled Amino Acid Transporter 4; *SLC38A2*, Sodium-coupled Neutral Amino Acid Transporter 2; *SLC38A9*, Sodium-coupled Neutral Amino Acid Transporter 9; *SLC7A1*, Cationic Amino Acid Transporter 1; *GAPDH*, Glyceraldehyde-3-Phosphate Dehydrogenase
